# Jin Fu Kang Oral Liquid Inhibits Lymphatic Endothelial Cells Formation and Migration

**DOI:** 10.1155/2016/3635209

**Published:** 2016-09-06

**Authors:** Hai-Lang He, Dan Wang, Jie Tang, Xian-Mei Zhou, Jian-Xin Li, Ling Xu

**Affiliations:** ^1^Department of Respiratory Medicine, Affiliated Jiangsu Province Hospital of Traditional Chinese Medicine, Nanjing University of Chinese Medicine, Nanjing 210029, China; ^2^State Key Laboratory of Analytical Chemistry for Life Science and Collaborative Innovation Center of Chemistry for Life Sciences, School of Chemistry and Chemical Engineering, Nanjing University, Nanjing 210023, China; ^3^Tumor Institute of Traditional Chinese Medicine, Shanghai University of Traditional Chinese Medicine, 725 South Wanping Road, Shanghai 200032, China; ^4^Department of Oncology, Yueyang Hospital of Integrated Traditional Chinese and Western Medicine, Shanghai University of Traditional Chinese Medicine, 110 Ganhe Road, Shanghai 200437, China

## Abstract

Lung cancer is the leading cause of cancer-related deaths worldwide. Jin Fu Kang (JFK), an oral liquid prescription of Chinese herbal drugs, has been clinically available for the treatment of non-small cell lung cancer (NSCLC). Lymphangiogenesis is a primary event in the process of cancer development and metastasis, and the formation and migration of lymphatic endothelial cells (LECs) play a key role in the lymphangiogenesis. To assess the activity of stromal cell-derived factor-1 (SDF-1) and the coeffect of SDF-1 and vascular endothelial growth factor-C (VEGF-C) on the formation and migration of LECs and clarify the inhibitory effects of JFK on the LECs, the LECs were differentiated from CD34^+^/VEGFR-3^+^ endothelial progenitor cells (EPCs), and JFK-containing serums were prepared from rats. SDF-1 and VEGF-C both induced the differentiation of CD34^+^/VEGFR-3^+^ EPCs towards LECs and enhanced the LECs migration. Couse of SDF-1 and VEGF-C displayed an additive effect on the LECs formation but not on their migration. JFK inhibited the formation and migration of LECs, and the inhibitory effects were most probably via regulation of the SDF-1/CXCR4 and VEGF-C/VEGFR-3 axes. The current finding suggested that JFK might inhibit NSCLC through antilymphangiogenesis and also provided a potential to discover antilymphangiogenesis agents from natural resources.

## 1. Introduction

Lung cancer, as the leading cause of cancer-related deaths worldwide, is the most common cancer affecting both men and women and holds approximately 27% of all cancer deaths in the United States [1]. Non-small cell lung cancer (NSCLC) accounts for >80% of all lung cancer cases [2]. The cancer cell migration to distant tissues occurs through blood and lymphatic vessels and is essential for tumor growth and metastasis [3]. Cancer metastasis is a very important event in cancer development and accounts for approximately 90% of treatment failure and related deaths for all cancer. However, effective approaches to inhibiting cancer metastasis have not yet been developed.

Lymphatic metastasis to regional lymph nodes has been focused on as a major indicator for the staging and the prognosis of most human cancers, and accurate lymph node staging is one of the most important factors in the NSCLC treatment and prognosis [4]. Growing evidences revealed that the lymphatic vasculature and tumors interact with each other and promote metastasis formation [5]. Lymphatic metastasis also closely relates to the tumor-induced formation and growth of new lymphatic vessels, named as lymphangiogenesis, an important initial event in tumor growth and spread [6]. Tumor-induced lymphangiogenesis plays a key role in promoting the initial spread of malignant tumor cells, and researches designed to block lymphangiogenesis are being carried out in the hope of arresting and reversing tumor development [7]. Therefore, the idea of blocking lymphangiogenesis might be a useful therapeutic strategy to restrict metastatic spread [8].

Lymphatic endothelial cells (LECs) play a vital role in regulation of lymphatic metastasis and lymphangiogenesis; inhibition of LECs formation and migration might reduce lymph node and organ metastasis [9, 10]. Circulating endothelial progenitor cells (EPCs) have the capacity to contribute to neovessel formation in the presence of proper stimuli [11]. Vascular endothelial growth factor-C (VEGF-C) and stromal cell-derived factor-1 (SDF-1 or CXCL12) are two critical factors in LECs formation and migration. VEGF-C stimulates cord blood-derived CD34 and vascular endothelial growth factor receptor-3-positive (CD34^+^/VEGFR-3^+^) EPCs to differentiate into LECs that express lymphatic vessel endothelial hyaluronan receptor-1 (LYVE-1), a lymphatic endothelial-specific marker [10]. Furthermore, although there are no direct evidences for the effect of SDF-1 on LECs formation and migration, SDF-1 closely relates to the tumor lymphangiogenesis and lymphatic metastasis [12]. Therefore, both VEGF-C and SDF-1 might be potential targets for therapeutic intervention on cancer [13].

Jin Fu Kang oral liquid (JFK), a Chinese herbal prescription, has been approved by China Food and Drug Administration and clinically available for the treatment of NSCLC [14, 15]. Studies have shown that JFK prevents tumor growth and progression and inhibits tumor angiogenesis in NSCLC patients. The possible mechanism might be via inhibition of the tumor cells to secrete VEGF [15, 16]. However, whether its antitumor effect correlates with inhibitory activity on LECs formation and lymphatic metastasis is still unclear.

In the present study, aiming at clarifying the activity of SDF-1 and coeffect of SDF-1 and VEGF-C on LECs formation and migration, CD34^+^/VEGFR-3^+^ EPCs were isolated from cord blood, and the LECs formation and migration were induced in the presences of SDF-1 and VEGF-C. Furthermore, the JFK-containing serums were prepared from JFK-dosed rats, and the inhibitory effects of JFK on formation and migration of LECs were investigated.

## 2. Materials and Methods

### 2.1. Animals and Ethics Statement

Twenty healthy male SD rats, weighing 200 ± 20 g, were purchased from the Experimental Animal Center of Zhejiang Province. The animals were housed in plastic cages with room temperature of 23 ± 1°C under a 12 h light-dark cycle and were given standard laboratory food and water freely. The rats were acclimatized for 3 days before the experiment.

The animal study was approved by the Jiangsu Animal Care and Use Committee and all of the protocols complied with the national and institutional rules regarding animal experiments.

### 2.2. Reagents

FITC-conjugated mouse anti-human CD34 and mouse IgG1 K isotype control-FITC were from eBioscience (CA, USA). APC-conjugated mouse anti-human VEGFR-3 and mouse IgG1 isotype control-APC were from R&D systems (USA). Anti-LYVE-1 antibody was from Abcam. FITC-conjugated secondary antibodies were from KeyGEN Biotechnology Company (Nanjing, China).

Ficoll-Hypaque solution was purchased from Haoyang Biotechnology Company (Tianjin, China). Goat serum and 4% paraformaldehyde were from Boster Biotechnology Company (Wuhan, China). VEGF-C and SDF-1 were from PeproTech. Lenvatinib (E7080) was from Selleckchem. AMD3100 was from Sigma-Aldrich. Dulbecco's modified Eagle's medium (DMEM) and fetal bovine serum (FBS) were from Hyclone. Complete growth medium EGM-2MV was from Lonza. 4′,6-Diamidino-2-phenylindole (DAPI) was from Beyotime Biotechnology Company (Shanghai, China). JFK (Lot number 130202) was obtained from Jilin Jinfukang Pharmaceutical Co., Ltd., (Jilin, China).

### 2.3. Preparation of Blank and JFK-Containing Serums

Twenty male SD rats were randomly divided into four groups: 5 rats per group. Rats in the blank group were given 3.6 mL/kg of distillated water; rats in the three JFK groups were orally given JFK at doses of 1.8, 3.6, and 7.2 g/kg three times per day for three successive days, respectively. After 1 h of final administration, the blood samples were collected from the abdominal aorta under ether anaesthesia. The blank or JFK-containing serums were acquired by centrifugation of the blood samples at 3000 rpm for 15 minutes at 4°C. All serum samples were then sterilized by suction with a 0.22 *μ*m Millipore filter and finally stored at −80°C for experiment use.

### 2.4. Collection of Mononuclear Cells from Cord Blood

Collection of cord blood was approved by Ethical Committee of Nanjing Health Hospital for Women and Children and performed from placentas of healthy delivery women. Mononuclear cells (MNCs) in cord blood were isolated by density-gradient centrifugation with ficoll solution at the density of 1.077. After centrifugation for 30 min at 400 g, the fraction of the mononuclear cells was collected. Then, the cells were gently washed with PBS in centrifugation for two times [17].

### 2.5. Flow Cytometry

For identification of CD34^+^/VEGFR-3^+^ cells, the mononuclear cells were incubated with combinations of FITC-conjugated mouse anti-human CD34 and APC-conjugated mouse anti-human VEGFR-3 for 30 minutes at 4°C; subsequently, the cells were washed twice with PBS. The immunoglobulin G isotype antibodies were used as negative controls. Then, the number of CD34^+^/VEGFR-3^+^ cells was determined with FACSort flow cytometer (Beckman Biosciences). All the staining was carried out according to manufacturer's protocols.

### 2.6. Sorting of EPCs from MNCs

For sorting of CD34^+^/VEGFR-3^+^ cells, the mononuclear cells were suspended with PBS containing 2% FBS and adjusted to 1 × 10^7^ cells/mL. After centrifugation, the cells were incubated with FITC-conjugated mouse anti-human CD34 and APC-conjugated mouse anti-human VEGFR-3 for 30 minutes at 4°C. Subsequently, the cells were washed with PBS containing 2% FBS and then resuspended with EGM-2MV medium in a 5 mL centrifuge tube [18]. CD34^+^/VEGFR-3^+^ cells were collected by cell sorting with a Beckman MoFlo*™* XDP FACS (fluorescence-activated cell sorter; Beckman Coulter, Fullerton, CA, USA).

### 2.7. Induction of Cell Differentiation

Firstly, the LECs were induced with SDF-1. The freshly sorted CD34^+^/VEGFR-3^+^ cells were suspended in EGM-2MV medium containing 60 ng/mL SDF-1, 20% FBS, 100 U/mL penicillin, and 100 *μ*g/mL streptomycin and seeded in a 24-well plate at a density of 1 × 10^5^ cells/well. The cells were incubated for 14 days in a humidified incubator at 37°C, 5% CO_2_, and the medium was changed every 3 days.

The LECs were also induced with VEGF-C and SDF-1 + VEGF-C with the same protocol as described above, except that VEGF-C (60 ng/mL) or SDF-1 (100 ng/mL) + VEGF-C (60 ng/mL) were used instead of SDF-1.

### 2.8. Transmission Electron Microscopy

After induction with SDF-1, VEGF-C, or SDF-1 + VEGF-C for 14 days, the differentiated cells were fixed with 2.5% glutaraldehyde overnight at 4°C and then postfixed with 1% osmium tetroxide. After being dehydrated with gradient alcohol, the cells were soaked with anhydrous acetone and Spurr resin and embedded with Spurr resin. Ultrathin sections were stained with 3% uranyl acetate and lead citrate [10]. The ultrastructural characteristics of the cells were examined by using a JEOL JEM-1010 transmission electron microscope.

### 2.9. Immunofluorescence

All the LECs differentiated from the CD34^+^/VEGFR-3^+^ cells in the presences of SDF-1, VEGF-C, or SDF-1 + VEGF-C and each JFK-containing serum were identified with expression of lymphatic endothelium specific markers, LYVE-1, as reported method [19]. Briefly, after induction for 14 days as described above, the cells were fixed by 4% paraformaldehyde and then incubated with rabbit anti-human LYVE-1 antibody (1 : 100) overnight at 4°C. After washing, the cells were incubated with FITC-labelled goat anti-rabbit IgG (1 : 200) for 60 minutes at 37°C. The nuclei were counterstained with DAPI. The cells were viewed by using confocal laser scanning microscope. Fluorescence images and integral optical density (IOD) values were further analyzed with Image-Pro Plus 6.0 software.

### 2.10. Transwell Migration Assay

The migration efficiency of LECs was assessed using 8 mm pore Transwell filter membrane (Corning) following reported method [20]. Briefly, the LECs were seeded at a density of 2 × 10^5^ cells/mL in the upper chamber, and in the lower chamber 100 ng/mL of SDF-1 was added. After incubation for 16 h, the cells on two sides of the membrane were dried in air and then fixed with 4% paraformaldehyde for 15 min. Subsequently, the cells were stained with DAPI for 10 min. The cells that migrated to the lower sides of the membrane were quantified by counting in 5 fields under a Nikon TE2000 inverted fluorescence microscope [21]. The same protocol was also performed in the presences of VEGF-C (60 ng/mL) or SDF-1 (100 ng/mL) + VEGF-C (60 ng/mL).

### 2.11. MTT Assay

To assess the cytotoxicity of the JFK-containing serums, MTT [3-(4,5-dimethylthiazol-2-yl)-2,5-diphenyl-2H-tetrazolium bromide] assay was performed. The CD34^+^/VEGFR-3^+^ cells solution (100 *μ*L) containing 10% (v/v) of the JFK serums was seeded in a 96-well plate at a density of 1 × 10^4^ cells/well and three parallel wells for each group. The cells were cultured for 48 h as described above. After that, 10 *μ*L of MTT dye solution (5 mg/mL in phosphate buffered saline pH 7.4) was added to each well and the plates were further incubated for 4 h. After incubation, the supernatant was aspirated and the formazan crystals were dissolved in DMSO and the optical density (OD) was measured at 490 nm.

### 2.12. Inhibition of JFK on LECs Formation

To test the effect of JFK, the CD34^+^/VEGFR-3^+^ cells were divided into 5 groups: control group, the cells were cultured with blank serum; JFK-1, JFK-2, and JFK-3 groups, the cells were cultured with JFK-containing serums prepared with JFK at doses of 5.4, 10.8, and 21.6 g/kg/day respectively, and 100 *μ*L of each serum was added in 0.9 mL of culture medium; and AMD group, the cells were cultured with AMD3100 (10 *μ*M), a SDF-1 inhibitor, as a positive control. SDF-1 (100 ng/mL) was added to all groups as described previously.

The same protocol as in the above was also conducted, in which VEGF-C (60 ng/mL) or SDF-1 (100 ng/mL) + VEGF-C (60 ng/mL) were used instead of SDF-1. Lenvatinib (10 *μ*M), a VEGF-C inhibitor, or AMD3000 + Lenvatinib (each 10 *μ*M) were used as positive controls, respectively.

### 2.13. Inhibition of JFK on LECs Migration

To test the effect of JFK-containing serums, Transwell migration assay was applied as described previously. In the lower chamber, SDF-1 (100 ng/mL) was added. The LECs were divided into 5 groups as described above and pretreated with the blank serum, JFK-containing serums (10%, v/v of culture medium), and AMD3000 (10 *μ*M), for 30 minutes, respectively.

The same protocol as in the above was also performed, and only VEGF-C (60 ng/mL) or SDF-1 (100 ng/mL) + VEGF-C (60 ng/mL) were added in the lower chamber instead of SDF-1. Lenvatinib (10 *μ*M) and AMD3000 + Lenvatinib (each 10 *μ*M) were used as positive controls.

### 2.14. Statistical Analysis

Data were expressed as means ± SD. Differences between experimental groups were assessed by the two-tailed *t*-test using SPSS 19.0 software. All analyses were performed using the SPSS 11.50 software package, and probability values of 0.05 or less were considered to be statistically significant.

## 3. Results

### 3.1. Preparation of JFK-Containing Serums

As described previously, JFK has been used for the treatment of NSCLC in China. JFK is made from 12 Chinese herbal drugs (Table 1). In order to get real effective compositions of JFK working in vivo, an animal experiment using rats was conducted to prepare JFK-containing serums. Three doses of 1.8, 3.6, and 7.2 g/kg (equivalent to raw herbal drugs) were applied for the serum preparations, and the dose of 3.6 g/kg was equal to clinical used one.

### 3.2. Sorting and Detecting of CD34^+^/VEGFR-3^+^ Cells

**Figure 1:**
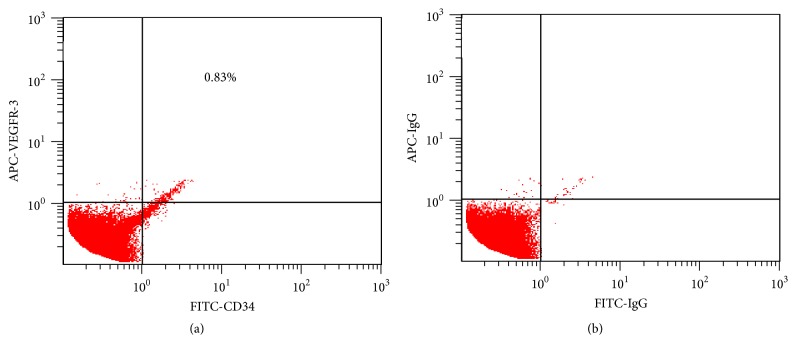
Sorting of CD34^+^/VEGFR-3^+^ cells. The mononuclear cells were analyzed for the expressions of CD34 and VEGFR-3 with dual-color flow cytometry (a), and percentage of the CD34^+^/VEGFR-3^+^ cells was compared with isotype control (b).

The mononuclear cells isolated from cord blood are round, having round-, ellipse-, or horseshoe-shaped nuclei. After being stained with FITC-conjugated mouse anti-human CD34 and APC-conjugated mouse anti-human VEGFR-3, the diameter of the cells coexpressing CD34 and VEGFR-3 was 10–12 *μ*m. By double-color flow cytometric analysis, the frequency of CD34^+^/VEGFR-3^+^ cells was 0.83 ± 0.21% in the mononuclear cells (Figures 1(a) and 1(b)), and the freshly sorted CD34^+^/VEGFR-3^+^ EPCs were round or oval.

### 3.3. SDF-1 and VEGF-C Additively Induced LECs Formation

**Figure 2:**
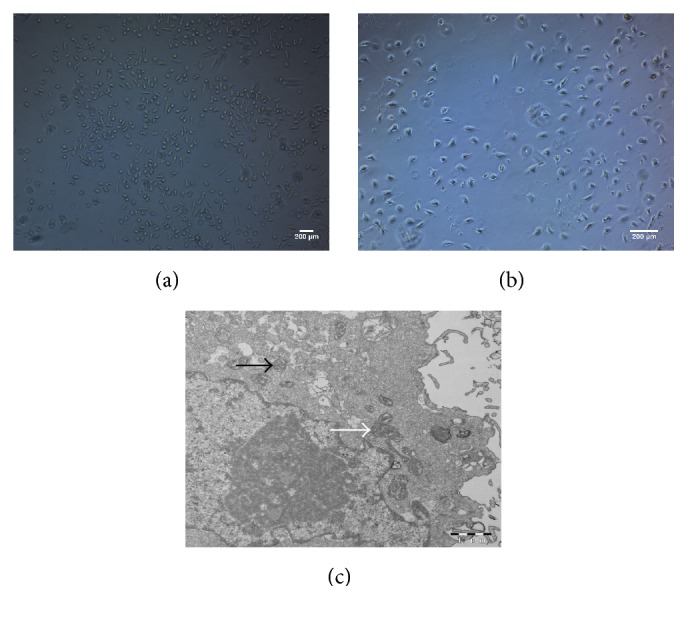
Morphological changes of CD34^+^/VEGFR-3^+^ cells during differentiation. At day 7 after induction with SDF-1, most cells became spindle-shaped (a). At day 14, the confluent monolayer of the cells demonstrated a typical cobblestone appearance (b). There are more mitochondria (white arrow) in the cytoplasm. Weibel-Palade body (black arrow) enwrapped by the membrane is observable (c).

Differentiation of CD34^+^/VEGFR-3^+^ cells towards LECs was firstly induced in the presence of SDF-1. As described above, the fresh sorted CD34^+^/VEGFR-3^+^ cells were round or oval, while, at day 3 after induction with SDF-1, some cells represented spindle shape or polygon shape. At day 7, the most cells were long spindle-shaped (Figure 2(a)). At the end of the experiment (day 14), the cells displayed polygonal shape and grew into confluent monolayer (Figure 2(b)).

To understand the ultrastructural characteristics of the cells, a transmission electron microscope examination was performed. The observation revealed that the nuclei of the cells were large, and there are more mitochondria and phagocytic vesicles in the cytoplasm. Furthermore, as can be seen in Figure 2(c), Weibel-Palade body, the unique rod-shaped secretory organelle of vascular endothelial cells, was clearly exhibited.

**Figure 3:**
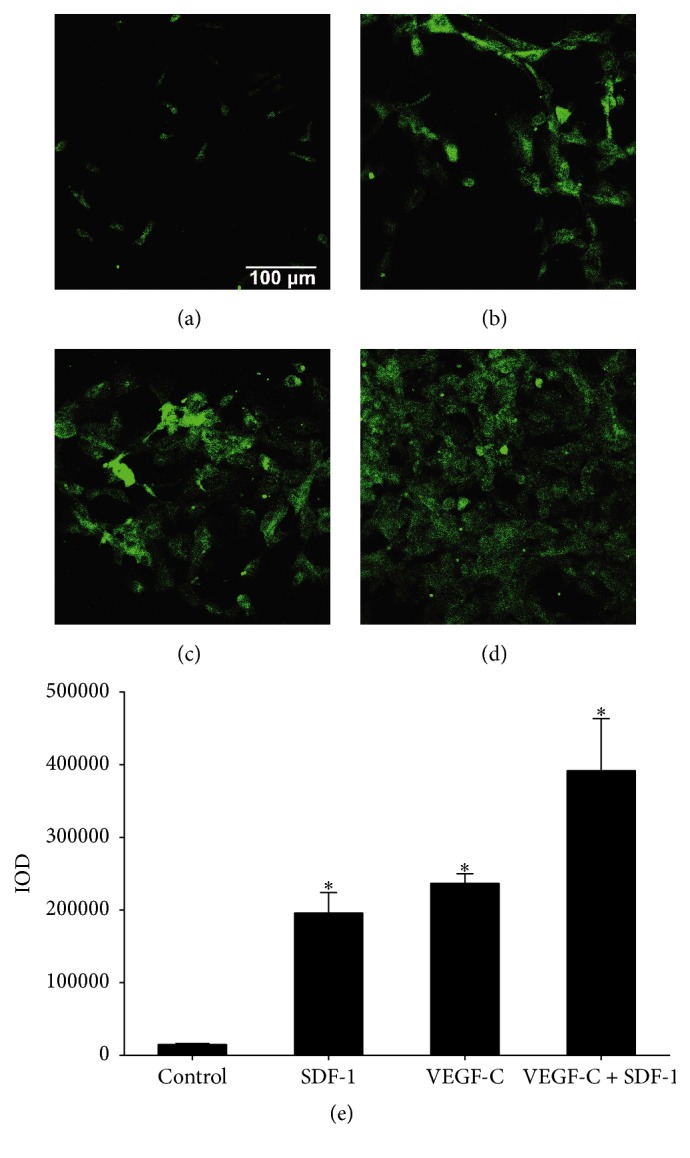
LECs with positive LYVE-1 immunostaining differentiated from CD34^+^/VEGFR-3^+^ EPCs. The CD34^+^/VEGFR-3^+^ cells were cultured in the presence of SDF-1 (100 ng/mL) and VEGF-C (60 ng/mL). The cells expressed LYVE-1 marker after induction with no addition ((a), control), SDF-1 (b), VEGF-C (c), and SDF-1 + VEGF-C (d) for two weeks. Integral optical density (IOD) of LYVE-1 fluorescence of each group (e). Values are expressed as mean ± SD, *n* = 3. ^*∗*^ *p* < 0.01, significant increase versus control group.

As lymphatic vessel endothelial hyaluronan receptor-1 (LYVE-1) is a specific marker of LECs, an immunofluorescence analysis was conducted with LYVE-1 antibody. The results showed that, after induction for 2 weeks in the presence of SDF-1, almost all of the cells were positive for LYVE-1 immunostaining (Figure 3(b)), while without SDF-1 stimulation (control), only a few LYVE-1 positive LECs were observed (Figure 3(a)).

VEGF-C was also used for the formation of LECs from CD34^+^/VEGFR-3^+^ cells. The result demonstrated that VEGF-C induced the differentiation of CD34^+^/VEGFR-3^+^ EPCs towards LECs, while the number of LECs was a little bit more compared with that of SDF-1 (Figure 3(c)).

As shown in Figure 3(d), the LYVE-1 positive cell number in SDF-1 + VEGF-C group was significantly greater than those of SDF-1 or VEGF-C alone. The integral optical density (IOD) value of LYVE-1 fluorescence in SDF-1 + VEGF-C group (391828) was almost the sum of those in SDF-1 (195995) and VEGF-C (236695) groups (Figure 3(e)). The results revealed that, compared with SDF-1 or VEGF-C alone, costimulation with SDF-1 and VEGF-C displayed an additive effect on induction of LECs formation.

### 3.4. SDF-1 and VEGF-C Promote Migration of LECs but Not Additively

**Figure 4:**
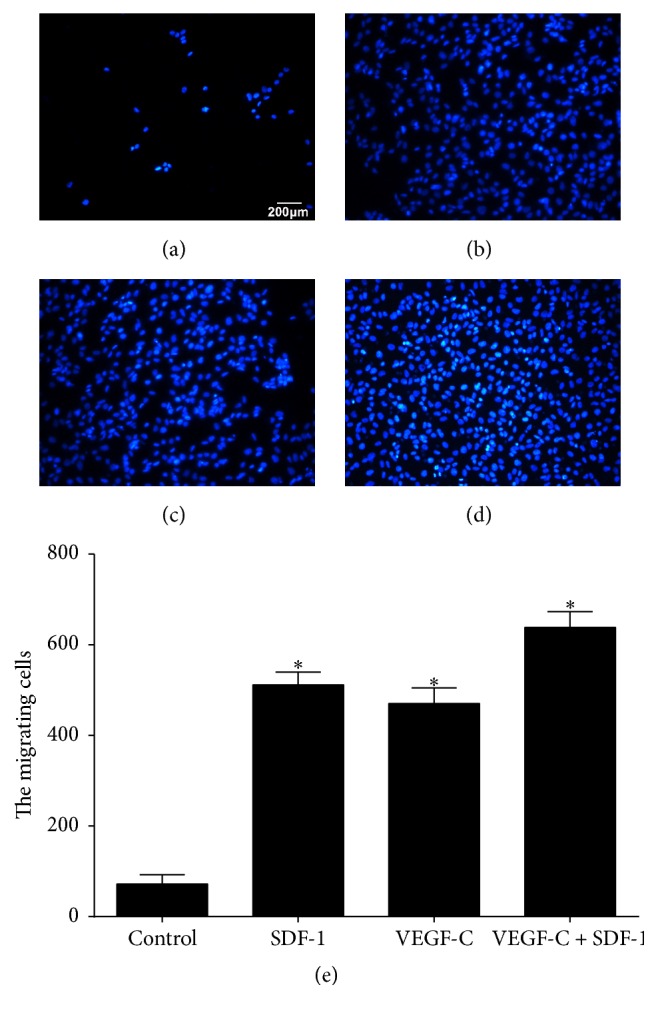
Transmigrated LECs in the lower chamber. In the lower chamber, no addition ((a), control), SDF-1 (100 ng/mL, (b)), VEGF-C (60 ng/mL, (c)), and SDF-1 (100 ng/mL) + VEGF-C (100 ng/mL) (d) were used. The migrated cells number in each group (e). Data are expressed as mean ± SD, *n* = 3. ^*∗*^ *p* < 0.01, significant increase versus control group.

In cell migration assay, LECs were seeded in the upper chamber of a Transwell insert, and firstly SDF-1 was added in the lower chamber. As shown in Figures 4(a) and 4(b), in the presence of SDF-1, the number of the LECs that migrated from the upper chamber to the lower chamber was significantly greater compared with that in no addition group. This result clearly indicated that SDF-1 induced the LECs migration. VEGF-C treatment also displayed the same effect as SDF-1 (Figure 4(c)).

However, when SDF-1 and VEGF-C were coused, although a little bit more LECs migrated to the lower chamber compared with those in SDF-1 or VEGF-C alone (Figures 4(d) and 4(e)), the cell number of SDF-1 + VEGF-C (638 cells) was far less than the sum of SDF-1 (511 cells) and VEGF-C (471 cells). The results clearly indicated that costimulation with SDF-1 and VEGF-C did not result in an additive effect on the cell transmigration.

### 3.5. Cytotoxicity of JFK-Containing Serums

**Figure 5:**
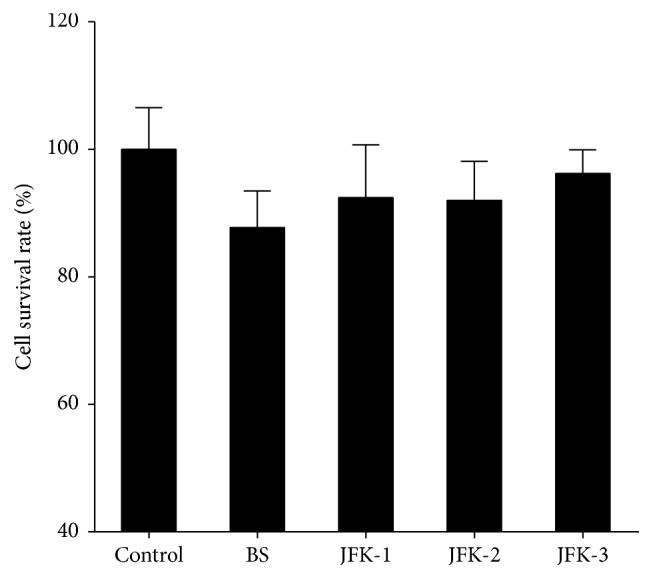
Cytotoxicity of JFK-containing serums. The EPCs were cultured with no addition (control), blank serum (BS), and JFK-1, JFK-2, and JFK-3. JFK-1, JFK-2, and JFK-3 represented the serums prepared from rats by oral administration of JFK at doses of 1.8, 3.6, and 7.2 g/kg, respectively, and 10% (v/v) of the serums were used.

To verify the cytotoxicity of JFK-containing serums, a MTT assay was performed. As shown in Figure 5, blank serum showed no effects on EPCs, and all the JFK-containing serum samples (10% concentration, v/v, serum to culture medium) exhibited no cytotoxicity on the CD34^+^/VEGFR-3^+^ EPCs during 2 days' culture period. Thus, the 10% concentration of JFK-containing serums was used for the following bioassays.

### 3.6. Inhibitory Effects of JFK on the LECs Formation

**Figure 6:**
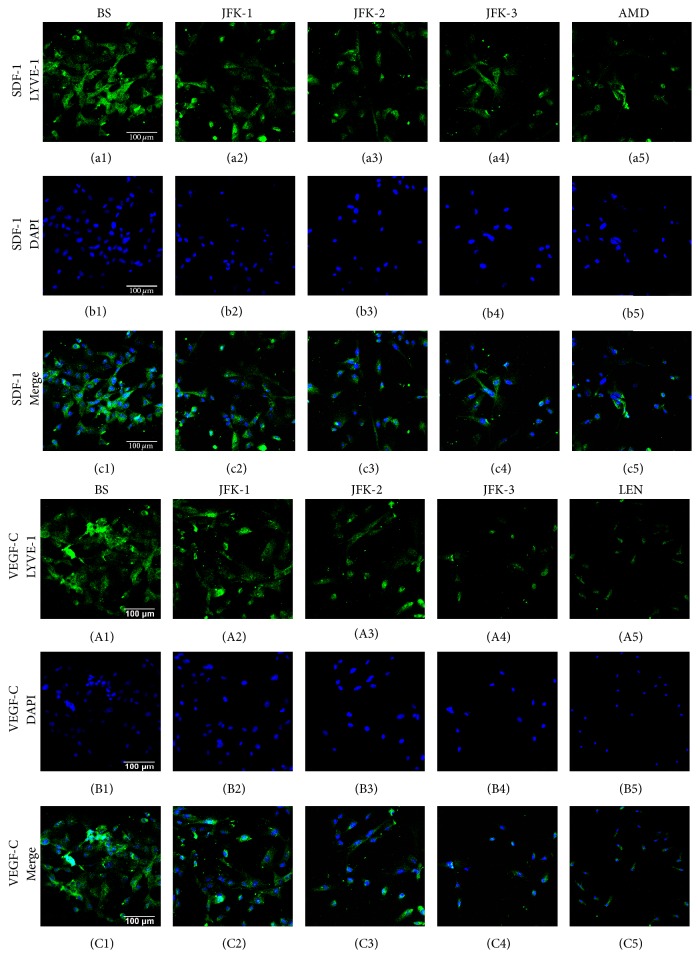
Effect of JFK on LYVE-1 positive LECs differentiation. The LECs formation was induced from CD34^+^/VEGFR-3^+^ EPCs with SDF-1 (100 ng/mL) and VEGF-C (60 ng/mL), respectively. BS, blank serum; JFK-1, JFK-2, and JFK-3, the serums prepared from rats by oral administration of JFK at doses of 1.8, 3.6, and 7.2 g/kg, respectively, and 10% (v/v) of the serums were added; AMD, AMD3100 (10 *μ*M); Len, Lenvatinib (10 *μ*M).

As JFK was used for the treatment of NSCLC, to understand whether JFK possesses the inhibitory effect on LECs formation, JFK-containing serums were tested. The result revealed that blank serum (BS) showed no impact on LECs formation (data not shown). As can be seen in Figure 6 and Table 2, in the positive control, AMD3100 (AMD), a SDF-1 inhibitor, significantly suppressed the IOD values of LYVE-1 about 93.73% compared with SDF-1 only (BS). As expected, all of the JFK-containing serums decreased IOD of LYVE-1; in particular, the serum of 7.2 g/kg reduced IOD value about 82.79%. These results suggested JFK-containing serums inhibited LECs formation.

The same as SDF-1, the LECs formations induced by VEGF-C and SDF-1 + VEGF-C were also suppressed by JFK-containing serums.

### 3.7. Inhibitory Effects of JFK on the LECs Migration

**Figure 7:**
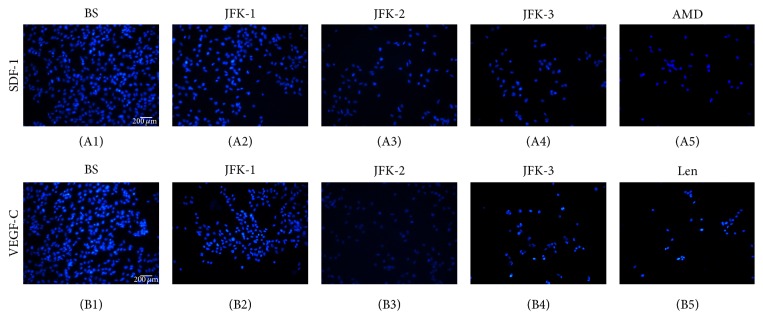
Effect of JFK on LECs transmigration. The LECs migration was induced with SDF-1 (100 ng/mL) and VEGF-C (60 ng/mL), respectively. The LECs were pretreated with blank serum (BS); the serums (each 10%, v/v) prepared from rats by oral administration of JFK at doses of 1.8, 3.6, and 7.2 g/kg (JFK-1, JFK-2, and JFK-3, resp.); AMD3100 (AMD, 10 *μ*M); Lenvatinib (Len, 10 *μ*M).

As tumor cell migration is an extremely important process in cancer metastasis, the effect of JFK was further evaluated on the LECs migration. As shown in Figure 7 and Table 3, the cell migration assay data clearly exhibited that JFK-containing serums prepared at all doses demonstrated a significant inhibitory effect on the SDF-1 and VEGF-C induced migrations. Furthermore, the LECs migration induced with SDF-1 + VEGF-C was also markedly suppressed by JFK-containing serums in a dose dependent manner. Blank serum showed no effect on the migration (data not shown). The SDF-1 and VEGF-C induced migrations were markedly blocked by their inhibitors, AMD3100 or Lenvatinib, respectively.

## 4. Discussion

In traditional bioassays in vitro on traditional Chinese medicines, generally, extracts or simply purified components of crude drugs are directly added to the culture system [22]. However, the pharmacological actions of traditional Chinese medicines are rather complicated due to their complex compositions. More important thing is that many of the components contained in the crud drugs do not directly exhibit their pharmacological actions until they experience a series of biotransformation during absorption, distribution, and metabolite procedures. Therefore, without biotransformation, the results from the bioassays in vitro might not truly reflect the pharmacological actions in vivo. Furthermore, the serum drug concentration directly reflects the drug efficacy in most cases. Therefore, serum pharmacology in which the drug-containing serum was prepared in vivo was used for bioactivity evaluation and research on traditional Chinese medicines in vitro, and the results were more scientific and exact compared with traditional pharmacology [23]. In the current study, the JFK-containing serums were prepared with rats and applied for the in vitro assays.

EPCs have been extensively studied as a potential marker for endothelial regeneration ability and shown to be a therapeutic potential by directly enhancing angiogenesis or forming new vessels through vasculogenesis. EPCs also exhibit a character to be highly migratory to ischemic areas [24]. It is reported that circulating CD34^+^VEGFR-3^+^ lymphatic/vascular endothelial progenitor cells correlate with lymph node metastasis in patients with epithelial ovarian cancer [25], and further study revealed that CD34^+^/VEGFR-3^+^ EPCs in human cord blood could differentiate into lymphatic endothelial cells [10]. Flow cytometry is widely used to quantify and study circulating EPCs, while EPC-specific markers are essential [11]. Therefore, in the current study, CD34 and VEGFR-3 were applied for the sorting of the target EPCs from human cord blood, and the results demonstrated that the frequency of the CD34^+^/VEGFR-3^+^ cells was 0.83% in the mononuclear cells, which was consistent with the reported data [18].

It is well accepted that LECs contribute to lymphangiogenesis that plays an active role in the induction of metastasis to the lymph nodes in cancers. CXC chemokine receptor 4 (CXCR4) plays a central role in tumor cell dissemination and metastasis development in more than 75% of all cancers [26]. SDF-1, also known as chemokine CXC ligand, binds to the CXCR4 receptor. The SDF-1/CXCR4 axis is essential for the migration of progenitor cells, tissue regeneration, and vascularization [27]. Furthermore, SDF-1/CXCR4 axis significantly associates with lymph node metastasis and promotes lymphangiogenesis in tumors [28]. VEGF-C/VEGFR-3 signaling is a key modulator of the lymphatic system, and VEGF-C level correlates with lymph node metastasis and poor prognosis [9]. VEGF-C promotes LEC proliferation, migration, and tube formation [10]. VEGFR-3 siRNA effectively inhibits differentiation and lymphangiogenesis of CD34^+^/VEGFR-3^+^ EPCs [29]. Therefore, in the present study, we used SDF-1 and VEGF-C to induce the LECs formation and migration. Our results clearly demonstrated that SDF-1 and VEGF-C promoted the CD34^+^/VEGFR-3^+^ EPCs to differentiate to the LECs that showed positive LYVE-1 staining, a specific LEC marker, and also enhanced the LECs migration.

Although it is reported that VEGF-C stimulation upregulates the expression level of CXCR4 on lymphangiogenic endothelial cells, SDF-1/CXCR4 axis exerts its function in a VEGFR-3 independent pattern [12]. More interestingly, targeting both SDF-1 and VEGF-C by neutralizing antibodies resulted in an additive inhibitory effect on both tumor lymphangiogenesis and lymphatic metastasis [12]. Furthermore, it is hypothesized that SDF-1 and VEGF may have synergetic effect on endothelial differentiation through a positive feedback loop with mutual promotion [30]. Thus, to clarify the coeffect of SDF-1 and VEGF-C, we added both in the culture system, and the data revealed that costimulation with SDF-1 and VEGF-C displayed an additive effect on the LECs differentiation; however, no additive effect was observed on the LECs migration, which needs a further mechanism clarification.

Studies have shown that JFK can prevent tumor growth and progression and inhibit tumor angiogenesis [16]. However, there is no data on whether JFK inhibits tumor lymphangiogenesis. The present work provided the first experimental evidence that JFK suppressed the formation and directional migration of LECs in vitro, which closely related to the tumor lymphangiogenesis. Furthermore, the inhibitory effect might be via regulation of SDF-1/CXCR4 and VEGF-C/VEGFR-3 axes. As JFK is composed of 12 herbal drugs, further effort on chemical isolation and structure identification of JFK is essential to clarify the bioactive components responsible for the effect and might provide a potential to discover the antilymphangiogenesis agents from the natural resources.

## 5. Conclusion

This study demonstrated that SDF-1 and VEGF-C induced CD34^+^/VEGFR-3^+^ EPCs to differentiate towards LECs and enhanced the LECs migration. SDF-1 and VEGF-C exerted an additive role on the LECs formation but not on the migration. JFK inhibited the differentiation and migration of LECs via regulation of the SDF-1/CXCR4 and VEGF-C/VEGFR-3 axes at least. The current finding suggested that JFK might inhibit NSCLC through antilymphangiogenesis and also provided a potential to discover antilymphangiogenesis agents. Further research on the inhibitory effect of JFK on lymphangiogenesis in vivo and clarification of its bioactive components are undergoing in our lab.

## Figures and Tables

**Table 1:** Composition of Jin Fu Kang oral liquid (JFK).

Herbal drug	Part used
*Astragalus membranaceus*	Root
*Glehnia littoralis*	Root
*Asparagus cochinchinensis*	Root
*Ligustrum lucidum*	Fruit
*Selaginella doederleinii*	Whole plant
*Paris polyphylla*	Root
*Epimedium sagittatum*	Leaf
*Gynostemma pentaphyllum*	Leaf
*Cornus officinalis*	Fruit
*Salvia chinensis*	Whole plant
*Ophiopogon japonicus*	Root
*Trigonella foenum graecum*	Seed

**Table 2:** Inhibitory effect of JFK on LECs formation.

Group	Inhibition rate (%)		
	SDF-1	VEGF-C	SDF-1 + VEGF-C
BS	0.00 ± 14.40	0.00 ± 5.61	0.00 ± 18.29
JFK-1	44.10 ± 6.80^*∗*^	38.89 ± 6.30^*∗*^	58.82 ± 12.37^*∗*^
JFK-2	60.57 ± 4.51^*∗*^	79.71 ± 1.31^*∗*^	76.06 ± 9.54^*∗*^
JFK-3	82.79 ± 6.44^*∗*^	89.14 ± 1.43^*∗*^	91.16 ± 2.31^*∗*^
AMD	93.73 ± 0.60^*∗*^		
Len		87.18 ± 5.80^*∗*^	
Len + AMD			96.33 ± 0.60^*∗*^

BS, blank serum; JFK-1, JFK-2, and JFK-3, the serums prepared from rats by oral administration of JFK at doses of 1.8, 3.6, and 7.2 g/kg, respectively; AMD, AMD3100; Len, Lenvatinib. The inhibition rates of BS groups were pegged as 0.00%, and others were calculated relative to it. Data were expressed as mean ± SD, *n* = 3. ^*∗*^ *p* < 0.01 compared with the BS group, respectively.

**Table 3:** Inhibitory effect of JFK on LECs migration.

Group	Inhibition rate (%)		
	SDF-1	VEGF-C	VEGF-C + SDF-1
BS	0.00 ± 5.47	0.00 ± 7.28	0.00 ± 5.52
JFK-1	44.19 ± 5.03^*∗*^	32.81 ± 11.36^*∗*^	34.96 ± 5.41^*∗*^
JFK-2	66.03 ± 2.82^*∗*^	69.87 ± 3.08^*∗*^	67.06 ± 5.49^*∗*^
JFK-3	80.49 ± 5.27^*∗*^	82.22 ± 5.70^*∗*^	72.04 ± 2.77^*∗*^
AMD	87.05 ± 3.79^*∗*^		
Len		84.69 ± 3.90^*∗*^	
Len + AMD			84.82 ± 2.09^*∗*^

BS, blank serum; JFK-1, JFK-2, and JFK-3, the serums prepared from rats by oral administration of JFK at doses of 1.8, 3.6, and 7.2 g/kg, respectively; AMD, AMD3100; Len, Lenvatinib. The inhibition rates of BS groups were pegged as 0.00%, and others were calculated relative to it. Data were expressed as mean ± SD, *n* = 3. ^*∗*^ *p* < 0.01 compared with the BS group, respectively.
